# Heat-treated high-fat diet modifies gut microbiota and metabolic markers in apoe−/− mice

**DOI:** 10.1186/s12986-016-0083-0

**Published:** 2016-03-12

**Authors:** Nittaya Marungruang, Frida Fåk, Eden Tareke

**Affiliations:** Food for Health Science Centre, Lund University, Medicon Village, Scheelevägen 2, SE-223 81 Lund, Sweden

**Keywords:** High-fat diet, Heat-treated high-fat diet, Advanced Glycation End products (AGEs), Gut microbiota, Atherosclerosis

## Abstract

**Background:**

High-fat diet has been known to have adverse effects on metabolic markers, as well as the gut microbiota. However, the effect of heat processing of high-fat diet, which leads to formations of advanced glycation end products (AGEs) has not been clearly distinguished from the effect of unheated fat. This study compared the effect of high-fat diet with heat-treated high-fat diet on adiposity, atherosclerosis and gut microbiota composition in the caecum of *apoe*^*−/−*^ mice.

**Method:**

Male *apoe*^*−/−*^ mice were fed either low-fat (LF) control diet, high-fat (40 E% saturated fat, HF) control diet, or heat-treated high-fat (200 °C for 10 min, HT) diet, for 8 weeks. The plasma samples were used in the analysis of Nε-carboxy-methyl-lysine (CML) and Nε-carboxy-ethyl-lysine (CEL). The heart samples were analysed for atherosclerotic plaques, and the DNA from caecum was extracted and analysed for microbiota composition using 16S rRNA gene sequencing on a Miseq instrument. Additionally, the functions of microbial communities were also predicted based on the bacterial 16S rRNA gene sequence using Phylogenetic Investigation of Communities by Reconstruction of Unobserved States (PICRUSt).

**Results:**

Here we found that HT modifies gut microbiota composition and host adiposity. Prediction of bacterial gene functions based on 16S rRNA gene sequence revealed that HF increased bacterial genera enriched in lipid metabolism genes, while HT did not. Plasma CML and CEL increased 1.7 and 2.5 times, respectively, in mice fed HT as compared to mice fed HF. Despite lower adiposity, mice fed HT maintained atherosclerosis and displayed enlarged spleens.

**Conclusions:**

The results suggested that heat processing of high-fat diet modifies the substrates reaching the lower gut of *apoe*^−/−^ mice, resulting in different effects on gut microbiota composition. AGEs seem to maintain the effect on atherosclerosis, despite lower adiposity, and causing enlarged spleens, which possibly reflect elevated levels of inflammation in the body.

**Electronic supplementary material:**

The online version of this article (doi:10.1186/s12986-016-0083-0) contains supplementary material, which is available to authorized users.

## Background

Modern diet involves heat processing of food, which leads to formation of Maillard reaction products (MRPs). These products are responsible for the aroma, colour, flavour, and texture as well as deterioration of nutritional value in the processed food and adverse health implications [1, 2]. Recently, more attention has been paid to the health implication of Maillard reaction products, especially those that lead to irreversible modifications in amino acids, peptides and proteins; advanced glycation end products (AGEs) [3–6]. AGEs are suggested to be associated to risk markers for inflammation mediated pathological conditions; such as cardiovascular disease, Alzheimer’s disease and diabetes-linked-complications [5, 7–12]. The mechanism for AGEs induced toxicity can be mediated through binding to AGE receptors (RAGE) and inducing pro-inflammatory cascade [8, 13, 14], and consequently associated pathological conditions. Cross link formation affecting the structure of the protein may also affect the digestibility and thus modified proteins may reach the lower gut where they can be fermented by gut microbiota [15].

The gut microbiota is intimately involved in numerous aspects of host physiology. The overall balance in the gut microbiota composition is an important key factor ensuring normal host functions [16]. Several studies have revealed that presence or absence of gut microbiota or some specific groups of bacteria have been found to contribute to development of many diseases such as type-2 diabetes [17, 18], artherosclerosis [19, 20] and systemic inflammatory response syndrome [21]. The composition of the gut microbiota is known to be affected by dietary components reaching the lower gut. So far, the role of process induced structure alteration in proteins on the digestibility and possible metabolism by gut microbiota is an area that is largely unexplored.

Atherosclerosis is one form of chronic inflammatory response to accumulation of fat in blood vessels. The apolipoprotein E knock out (*apoe*^−/−^) mouse is an animal model used for assessing effects of nutritional agents on lipid metabolism, inflammation and atherosclerosis [22]. *Apoe−/−* mice have an impaired ability for clearance of plasma lipoprotein, leading to the development of atherosclerosis in a short time. Several studies have been focusing on the pathological effects of high-fat diet. High intake of dietary fat has been known to induce several dynamic metabolic alterations, especially atherosclerosis, as well as changes in gut microbiota composition [17]. However, the fact that intake of fat is usually accompanied by heat processing [23], the specific effect of high fat intake versus the effect of AGEs has not been clearly distinguished. In the present study, we aimed to compare the effect of high-fat diet (40 E% saturated fat) with heat-treated (200 °C for 10 min) high-fat diet on adiposity, atherosclerosis and gut microbiota composition in the caecum of *apoe*^*−/−*^ mice. Moreover, the study compared the possible effect of intake of high-fat diet on the above mentioned end points by comparing with intake of low-fat diet. Additionally, the functions of microbial communities were also predicted based on the bacterial 16S rRNA gene sequence using a recently developed software, Phylogenetic Investigation of Communities by Reconstruction of Unobserved States (PICRUSt) [24].

## Methods

### Experimental design

Male *apoe*^*−/−*^ mice (Scanbur AB, Karlslunde, Denmark), 6 weeks of age, were adapted to the environment at the animal facility for 2 weeks before starting the experiment. At the age of 8 weeks, the mice were randomly divided into three weight-matched groups (*n* = 10, five mice/cage). The mice were fed low-fat (LF) control diet, high-fat (HF) control diet, or heat-treated high-fat (HT) diet. A picture of HF and HT is available as Additional file 1: Figure S1. The mice were weighed every week. After 8 weeks, the mice were anesthetized with isofluorane (Abbott Scandinavia AB, Solna, Sweden) and terminated by heart puncture. The liver, spleen and epididymal fat pads were weighed. Blood plasma, heart, and caecum were collected and frozen at −40 °C until further analyses. The plasma samples were used in the analysis of CML and CEL. The heart samples were analysed for atherosclerotic plaques, and the DNA from caecum was extracted and analysed for microbiota composition using 16S rRNA gene sequencing on a Miseq instrument.

### High performance liquid chromatography coupled to tandem mass spectrometry for determination of CML and CEL

Plasma samples (50 μL) were hydrolysed for 12 h at 110 °C using 6 M HCl, together with isotope labelled d4-CML and d4-CEL as internal standard (Larodan Fine Chemicals AB, Malmö Sweden), and analysed using high-pressure liquid chromatography mass spectrometry (HPLC-MS/MS). The chromatographic separation of CML and CEL in the hydrolysed samples was performed using Accela UHPLC pump with auto-injector. Detection was performed using a LTQ VelosPro Orbitrap massspectrometer (Thermo Scientific, Waltham, USA) run in positive electrospray ionization ion trap tandem mass spectrometry (MS/MS) mode, detecting two selective-reaction monitoring (SRM) transitions for each analyte, and internal standard. The Xcalibur software (Thermo Scientific) was used both for data acquisition and evaluation. Solid phase extraction, chromatographic parameters, ion source parameter and the SRM transitions are the same as described in Tareke E. et al. [25].

### Quantification of atherosclerosis

Frozen 10 μm sections of the aortic root region of the heart were prepared using a cryostat (Leica CM 1950, Leica Biosystems, Nusslock, Germany) and stained with Oil Red O (Histolab, Gothenburg, Sweden) and haematoxylin (Mayer’s HTX, Histolab, Gothenburg, Sweden). Amount of atherosclerotic plaques covering the vessel area was quantified by a blinded observer using BioPixQ 2.0 (Biopix software, Gothenburg, Sweden). Three sections/mouse, with well-oriented root regions and all three valve cusps present, were evaluated.

### Caecal microbiota composition

A total of 22 caecal samples (LF (*n* = 4), HF (*n* = 8), HT (*n* = 9)) were used in the microbiota analysis. Caecal tissue and content were thawed on ice and DNA was extracted using the QIAamp DNA Stool Mini Kit (Qiagen), with an addition of a bead beating step. Sterile glass beads (1 mm) were added in combination with stool lysis buffer and cell disruption was performed for 2 × 2 min at 25 Hz using a TissueLyser (Qiagen), followed by a heating step at 95 °C for 5 min. After lysis, DNA-damaging substances and PCR inhibitors were removed using InhibitEX tablet (provided with the kit) and the DNA was purified on QIAamp Mini spin columns. A normalized input of 5 ng/μL of the DNA was used in PCR reactions where 16S rRNA genes were amplified prior to sequencing.

The V1-V3 regions of 16S rRNA genes were amplified using a limited cycle PCR with forward and reverse primers containing Illumina adapter sequences and dual-index barcodes used for tagging each sample, primer sequences are listed in Table 1. Paired-end sequencing with a read length of 2×300 bp was performed on a Miseq Instrument (Illumina, Inc, San Diego, California) using a Miseq v3 reagent kit (Illumina, Inc, San Diego, California). Sequences were analysed using Quantitative Insights into Microbial Ecology (QIIME), as previously described [26]. The Operational Taxonomic Units (OTUs) were picked using a closed-reference OTU picking with UCLUST [27] algorithm against the Greengenes [28] database (version 13_8), pre-clustered at 97 % identify. After quality filtering, a total of 2,280,567 reads were included for downstream analyses and an average of 103,662 sequences (SD: 18,962) were assigned to each sample (ranging from 67,817 to 145,015 sequences). The relative abundances of bacterial taxa at phylum and genus levels were thereafter compared between groups. To correct for sampling depth differences, 67,817 reads/sample were randomly selected and utilized for further calculation of alpha-diversity and weighted and unweighted Unifrac as well as correlations between the gut microbiota and different metabolic markers.

**Table 1:** Primer sequences for amplification of 16S rRNA genes, amplicon length 507 bp

16S Amplicon PCR Forward Primer	27F AGAGTTTGATCCTGGCTCAG
16S Amplicon PCR Reverse Primer	534R ATTACCGCGGCTGCTGG

### Prediction of bacterial metagenomes using PICRUSt

The bacterial metagenomes were reconstructed based on the bacterial 16S rRNA gene sequence using the open-source software, PICRUSt [24]. The OTU table generated in QIIME from the 16S sequencing data at 67,817 rarefied sequences per sample was used as an input. The copy number per OTU was normalized before the metagenome was predicted using Kyoto Encyclopedia of Genes and Genomes (KEGG) database [29]. The output from metagenome prediction was an annotated table of predicted gene family counts for each sample, where gene families were grouped by KEGG Orthology (KO) identifiers.

### Statistical analyses

One-way analysis of variance (ANOVA) was used to calculate the differences in metabolic markers in different groups of mice using Graphpad Prism 6. Differences in within-community richness (α-diversity) were calculated in QIIME using a non-parametric *t*-test and the *P*-values were corrected for multiple comparisons using False Discovery Rate (FDR) correction [30]. Differences in community composition among groups of samples (β-diversity) were analyzed using the non-parametric analysis of similarity (ANOSIM) [31] statistical test in QIIME on both unweighted- and weighted Unifrac phylogenetic metrics. Graphpad Prism 6 was also used to identify significant differences in taxonomic distributions at phylum and genus levels between the groups of mice using two-way ANOVA and the Holm-Sidak method of correction for multiple comparisons. All the differences were considered to be significant at *P* < 0.05. Moreover, linear discriminant analysis (LDA) effect size (LEfSe) analysis [32] was performed to identify differentially abundant bacterial taxa (from phylum to species level) as well as the predicted metagenomes in each group of mice. A strict version of LEfSe was used, the discovered bacterial taxa and predicted genes with LDA score higher than two were considered to be enriched in the respective group as compared to all other groups. Partial Least Squares (PLS) Regression analysis was performed using SIMCA14 (Umetrics, Umeå, Sweden) and PLS loading score scatter plots were plotted to illustrate the associations between the parameters. Pearson’s correlation was calculated for each pairwise combination of bacterial genera versus relative spleen weight, plasma CEL and CML, and atherosclerosis plaque using Graphpad Prism 6 and the *P*-values were then corrected by Benjamini-Hochberg procedure for multiple comparisons [30, 33].

## Results

### Biomarkers

**Fig. 1:**
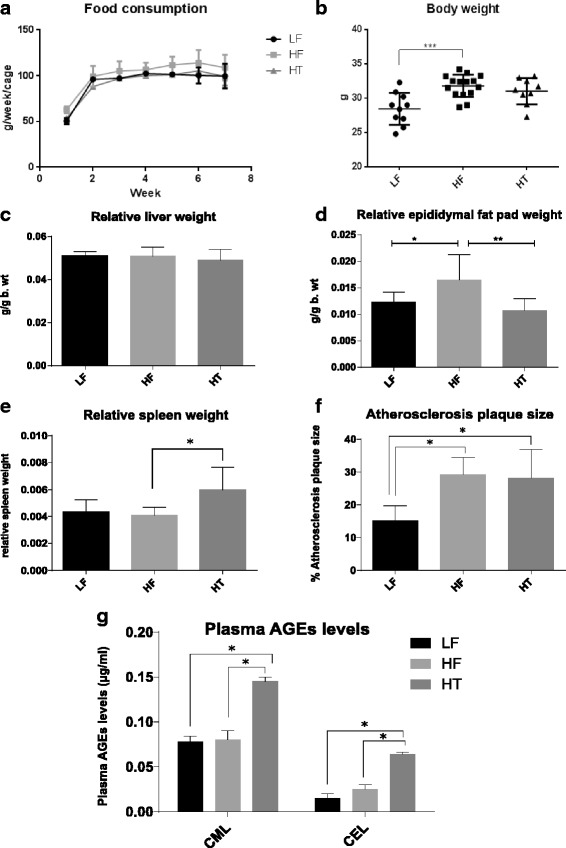
Different biomarkers in mice fed LF, HF and HT. **a** Food consumption (g/week/cage). **b** Mice body weight at end point (g). **c** Relative liver weight (g/g body weight). **d** Relative epididymal fat pad weight (g/g body weight). **e** Relative spleen weight (g/g body weight). **f** Percentage of atherosclerotic plaque size (%). **g** Plasma levels of AGEs products, CML and CEL (μg/ml). One-way ANOVA was used to calculate significance among the different groups (*P < 0.05, **P < 0.01)

Considering no difference in food consumption of the mice in different groups (Fig. 1a), mice fed HF had significantly higher body weight at the end point as compared to mice fed LF (*P* < 0.001, Fig. 1b). The liver weight did not differ between groups (Fig. 1c), while the epididymal fat pad weight was significantly lower in the HT and LF groups compared to the HF group (Fig. 1d). Relative spleen weight was significantly higher in HT mice as compared to HF mice (*P* < 0.05, Fig. 1e). Atherosclerotic plaque size (% of vessel area) was higher in HF and HT as compared to LF (both *P* < 0.05, Fig. 1f). Analysis of Advanced glycation end products (CML and CEL levels) in plasma of mice revealed significant increases in levels of CML and CEL in plasma of HT mice as compared to LF and HF (all *P* < 0.05, Fig. 1g). CML increased 1.7 times in HT mice as compared to LF and HF, while CEL increased 3.5 and 2.5 times in HT as compared to LF and HF, respectively. There was no significant difference in plasma CML and CEL levels between HF and LF mice.

### Microbial diversity indices

**Fig. 2:**
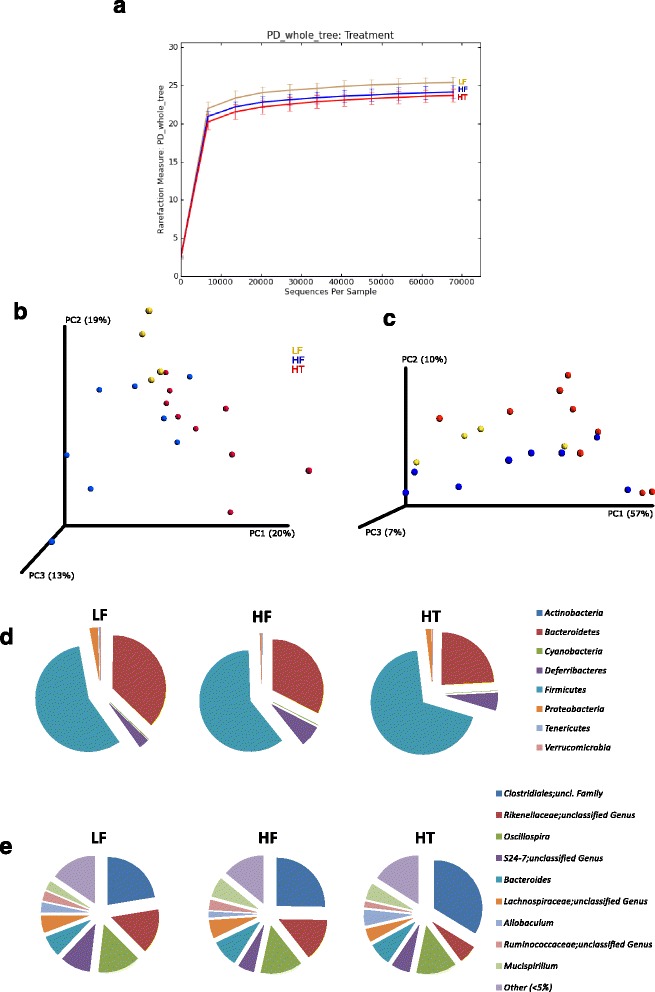
Diversity indices and distributions at phylum and genus levels of the gut microbiota in mice fed LF, HF and HT. **a** Alpha rarefaction curve (whole-tree PD) showing differences in within-community richness (α-diversity). **b** Unweighted and **c** Weighted UniFrac PCoA plots showing differences in community composition among groups of mice (β-diversity). **d** Relative abundance of the gut microbiota at phylum. **e** Relative abundance of the most abundance genera (>5 % relative abundance). Reads normalized to 67,817 sequences/sample

Estimation of α-diversity of bacterial 16S rRNA gene at the sequence number of 67,817 sequences/sample (Fig. 2a) demonstrated that HF had a tendency for decreased α-diversity as compared to LF. Moreover, HT lowered the α-diversity even more as the observed OTUs and phylogenetic diversity whole tree (PD whole-tree) indices showed significant differences between HT and LF (*P* < 0.05), although there was no significant difference with Chao1 index. ANOSIM analyses of both unweighted and weighted Unifrac revealed significant differences between the communities of the mice in the three treatment groups (*P* < 0.01 and *P* < 0.05, respectively). Principle coordinate analysis (PCoA) plots for unweighted and weighted Unifrac are shown in Fig. 2b and c, respectively.

### Phylum-level taxonomic distributions

Relative abundance at phylum-level (Fig. 2d) revealed that Firmicutes was found to be the most dominant phylum in all groups and was followed by Bacteroidetes. The level of Firmicutes was significantly higher in HT than LF and HF (both *P* < 0.01), whereas the level of Bacteroidetes was significantly lower in HT than LF and HF (*P* < 0.001 and *P* < 0.01, respectively).

### Genus-level taxonomic distributions

The most abundant genera presented in the mice (more than 5 % relative abundance) are shown in Fig. 2e. As compared to LF, HF significantly increased *Mucispirillum* (*P* < 0.05) and unclassified family of *Clostridiales* (*P* < 0.05) and reduced unclassified genus of *S24-7* (*P* < 0.001), while similar effects were observed in HT with significant increase in the relative abundance of unclassified family of *Clostridiales* (*P* < 0.001) and significant decrease in the relative abundance of unclassified genus of *S24-7* (*P* < 0.01)*.* Moreover, a significant decrease of an unclassified genus of *Rikenellaceae* (*P* < 0.001) was also observed in HT, as compared to LF. In addition, HT was found to reduce the relative abundance of an unclassified genus of *Rikenellaceae* (*P* < 0.001) as well as to increase the relative abundance of *Allobaculum* (*P* < 0.001) and unclassified family of *Clostridiales* (*P* < 0.001) as compared to HF.

### Differences in bacterial composition between groups of treatment using LEfSe

**Fig. 3:**
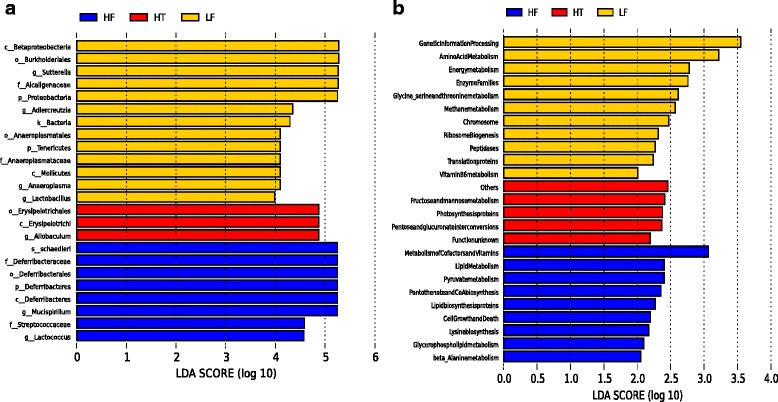
LDA score plot of bacterial taxa (**a**) and genes (**b**) with LDA scores higher than 2. Bacterial taxa and genes enriched in LF are in *yellow*, HF in *blue* and HT in *red*

The strict version of LEfSe (all against all) detected 24 bacterial clades showing statistically significant differences between different groups of treatment with LDA scores higher than two (Fig. 3a). A Cladogram of these bacterial taxa is shown in Additional file 2: Figure S2. The most enriched bacteria in LF were *Sutterella* (from phylum to genus), *Anaeroplasma* (from phylum to genus), *Adlercreutzia* and *Lactobacillus. Mucispirillum schaedleri* (from phylum to species) and *Lactococcus* (from family to genus) were found to be the most enriched bacteria in HF while *Allobaculum* (from order to genus) was found to be enriched in HT.

### Prediction of functional structure of genes based on 16S gene information using PICRUSt

By applying PICRUSt to the 16S sequencing data, a total of 328 genes were predicted to be involved in different functions (data not shown). Assigning the strict version (all against all) of LEfSe to the predicted genes from PICRUSt, detected a total of 11, 9 and 5 genes enriched in LF, HF and HT, respectively, with LDA scores higher than two (Fig. 3b). Of these, 7, 8 and 4 genes, enriched in LF, HF and HT, respectively, were involved in metabolism. Predicted metabolism genes enriched in LF encoded for metabolism of two amino acid groups; arginine and proline (KO00330), glycine, serine and threonine (KO00260), energy metabolism, particularly for methane metabolism (KO00680), enzyme families, especially for peptidases, and cofactors and vitamins, especially for vitamin B6 (KO00750). Predicted genes encoding for lipid metabolism, especially for lipid biosynthesis proteins (KO01004) and glycerophospholipid (KO00564) were enriched in HF. Moreover, metabolism of pyruvate (KO00620), lysine biosynthesis (KO00300), beta-alanine (KO00410), as well as pantothenate and CoA biosynthesis (KO00770) were also enriched in HF. These genes enriched in HF were shown to be involved in a cascade of the fatty acid biosynthesis KEGG pathway. Predicted genes encoding for carbohydrate metabolism; fructose and mannose metabolism (KO00051) and pentose and glucuronate interconversions (KO00040), energy metabolism encoding for photosynthesis proteins and an unclassified metabolism (no KO number) were enriched in HT.

Additionally, genes enriched in LF were involved in genetic information, especially for replication and repair of chromosome, ribosome biogenesis and an unclassified translation protein. A gene enriched in HF was involved in cellular processes, especially for cell growth and death and a gene enriched in HT was involved in an unclassified function (Fig. 3b).

### Gut microbiota and metabolic biomarkers

**Fig. 4:**
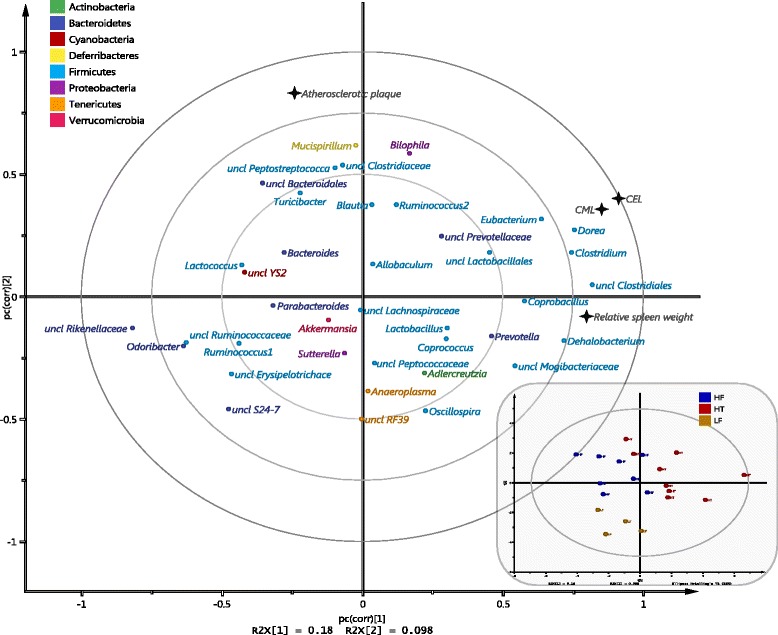
Loading (*big panel*) and score scatter (*small panel*) PLS plots showing correlations between the gut microbiota and different biomarkers. Bacterial genera are shown in small circles and colored by the phylum they belong to (*green*, *Actinobacteria*; *dark blue*, *Bacteroidetes*; *red*, *Cyanobacteria*; *yellow*, *Deferribacteres*; *light blue*, *Firmicutes*, *purple*, *Proteobacteria*; *orange*, *Tenericutes*; *pink*, *Verrucomicrobia*). Metabolic biomarkers are shown in black stars. Each circle in score scatter plot (small panel) represents each mouse and is colored by the group they belong to (*yellow*, LF; *blue*, HF; *red*, HT)

**Fig. 5:**
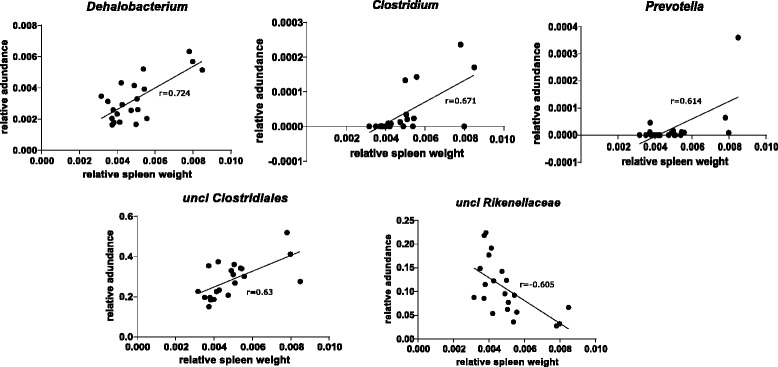
Linear regression plots with Pearson’s correlation coefficient (r) of bacterial genera significantly correlated with relative spleen weight. *Dehalobacterium*, *Clostridium, Prevotella*, unclassified family of *Clostridiales* and unclassified genus of *Rikenellaceae* were significantly correlated with relative spleen weight. Pearson’s correlations with *P* < 0.05 after correction for multiple comparison using Benjamini-Hochberg procedure was considered to be significant

Loading and score scatter (Fig. 4) PLS plots illustrated associations between the gut microbiota and different biomarkers as well as revealed separate clusters of the mice by different treatments (Fig. 4, small panel). The results supported the LEfSe results (Fig. 3a and Additional file 2: Figure S2) where those bacteria enriched in different groups of treatment were located in the corresponding area of the PLS plot (Fig. 4). *Sutterella, Anaeroplasma, Adlercreutzia* and *Lactobacillus* which were found to be enriched in LF were located in the lower component of the PLS plot. *Mucispirillum* and *Lactococcus* enriched in HF were located in the upper right component, while *Allobaculum* was located in the upper left component of the PLS plot. Correlations between the gut microbiota and different biomarkers were also analyzed. Relative spleen weight was found to be positively correlated with *Dehalobacterium* (*P* < 0.01), *Clostridium* (*P* < 0.05)*, Prevotella* (*P* < 0.05), unclassified family of *Clostridiales* (*P* < 0.05) and negatively correlated with unclassified genus of *Rikenellaceae* (*P* < 0.05) (Figs. 4 and 5). Plasma CEL and CML levels were located in the right component of and close to the relative spleen weight on the PLS plot. These suggested positive correlations between plasma CML and CEL versus relative spleen weight, and thus versus those bacterial genera that correlated with spleen weight, although the correlations were not statistically significant after correction for multiple corrections. No correlation was found between the gut microbiota and amount of atherosclerotic plaques.

## Discussion

The findings from this study revealed that heat processing of fat led to changes in its effects on metabolic markers and the gut microbiota in *apoe*^−/−^ mice as compared to unheated fat. Mice fed HT displayed elevated circulating levels of CML and CEL as well as enlarged spleens, to a higher extent than mice fed HF. Enlargement of the spleen has previously been observed even in healthy mice with intra-peritoneal administration of RAGE and this has been suggested to be exerted through stimulation of adaptive immune system in response to inflammation [34]. Interestingly, the amounts of atherosclerotic plaque were similar in HF and HT, which were both higher than LF. The amount of atherosclerotic plaques was anticipated to be higher in mice fed HT as heat processing of fat would add up the effect of AGEs to the effect of fat on atherosclerosis. The lower epididymal fat-pad weight observed in HT mice, to the same level as in LF mice, could possibly provide an explanation. Chemical modifications of fat by heat-treatment might abate the effect of unheated fat, leaving only the effect of AGEs on atherosclerosis. Thus, HT mice reached similar levels of atherosclerosis as HF mice despite lower adiposity, which may suggest an additive effect of AGEs on atherosclerosis in the HT mice. Heat-treated LF has not been included in this study as the aim was only to compare the effects of fat and heat-treatment of HF (HT), using LF as a baseline control. However, heat-treatment of LF diet may provide more insight into the role of fat and AGEs on atherogenesis.

The results from 16S sequencing of caecal samples showed that HF and HT had an impact on the composition of the gut microbiota in the mice. Both HF and HT lowered the diversity of the gut microbiota, although the change was only significant with HT. The most dominant phyla in all groups were Firmicutes and Bacteroidetes, whose relative abundance were significantly altered with HT, as well as with HF but to a lesser extent. An imbalanced ratio of Firmicutes and Bacteroidetes has been found to be associated with risk factors of obesity in both animals and humans. High dimensional comparison using LEfSe analysis showed that both HF and HT mice differed in their gut microbiota composition and functions, as compared to each other and to LF mice. The LF group had a high diversity of bacterial genera from different phyla. Although most groups of bacteria found enriched in LF are not well known regarding their roles for the host, the predicted gene functions revealed increases of genes involved in a broad range of metabolism pathways including amino acids, enzymes, as well as cofactors and vitamins. *Mucispirillum* found enriched in the mice fed HF, is one of the putative mucin degraders [35] and has been found in a study of Belzer et al., to be involved in the onset of symptomatic colitis in mice [36]. Interestingly, the lactic acid bacteria, *Lactococcus,* found enriched also in the mice fed HF, has been found in a study of Parks et al., to have positive correlation with body fat percentage gain in mice fed high-fat, high-sucrose diet [37]. Although genes enriched in HF mice were encoded for several metabolism pathways, it is remarkable that most of these were involved in a cascade of a fatty acid synthesis pathway. Some bacteria have been known to synthesize glycerophospholipid on the inner leaflet of the inner membrane and different lipid biosynthetic mechanisms have previously been defined [38]. These bacterial genera could therefore generate even more fat to the host’s energy metabolism. In addition, the gut microbiota composition has also been shown to play a role in how much energy is harvested from the diet [39–41], and indeed, mice fed HF had significantly higher epididymal fat pad weight, as well as slightly higher body weight than mice fed HT. Interestingly, *Allobaculum* which has previously been found to be decreased in mice fed high-fat diet [37, 42], became enriched in HT mice in this study. Although these studies showed somewhat similar 16S gene results as observed in this current study, it is worth mentioning that some of them were performed on animal faeces as opposed to caecum and these two locations in the gut may harbour different bacterial species. Together with the finding that no bacterial genes encoding for lipid metabolism were enriched in mice fed HT, we hypothesize that heat processing of fat changed the chemical properties of the fat, allowing different substrates to reach the lower gut. However, the exact mechanism of how heat-treated fat appears to be less accessible to the gut microbiota than unheated fat needs further investigation.

## Conclusions

In conclusion, intake of high-fat diet led to increased adiposity, decreased microbiota diversity and altered gut microbiota composition. Chemical modifications of high-fat diet by heat processing seemed to reduce the substrates available for members of the gut microbiota having lipid metabolism genes, resulting in lower adiposity. However, the overall alterations of the gut microbiota are still towards those associated with risk factors of obesity. Moreover, AGEs formed in HT could be a possible cause of spleen enlargement, which possibly reflect elevated levels of inflammation in the body. Lower intake of heat-processed food may need to be taken into consideration when developing a dietary preventative approach of the metabolic syndrome.

### Ethics approval

The study was approved by the local ethical review committee for animal experiments in Lund, Sweden (approval number M-295-12).

## Additional files

Additional file 1: Figure S1. Unheated (left) and heat-treated (200 °C for 10 min, right) high-fat diets. (PDF 241 kb) Additional file 2: Figure S2. Cladogram plot showing bacterial taxa with LDA scores higher than two using strict version of LEfSe. Different colors represent the most abundant taxa in different groups of mice (yellow indicating LF, red indicating HF and blue indicating HT). Circles represent phylogenetic levels from phylum to genus. The sizes of the circle are proportional to the taxon’s abundance. (PDF 436 kb)

## Abbreviations

AGEs: Advanced Glycation End products
ANOSIM: analysis of similarity
ANOVA: analysis of variance
apoe−/−: apolipoprotein E knock out
CEL: Nε-carboxy-ethyl-lysine
CML: Nε-carboxy-methyl-lysine
DNA: deoxyribonucleic acid
HF: high-fat diet
HPLC-MS/MS: high-pressure liquid chromatography mass spectrometry
HT: heat-treated-high-fat diet
KEGG: Kyoto Encyclopedia of Genes and Genomes
KO: Kyoto Encyclopedia of Genes and Genomes Orthology
LDA: linear discriminant analysis
LEfSe: linear discriminant analysis effect size
LF: low-fat diet
MRPs: Maillard reaction products
MS/MS: tandem mass spectrometry
OTU: operational taxonomic unit
PCoA: principle coordinate analysis
PCR: polymerase chain reaction
PD: phylogenetic diversity
PICRUSt: Phylogenetic Investigation of Communities by Reconstruction of Unobserved States
PLS: Partial Least Squares
QIIME: Quantitative Insights into Microbial Ecology
RAGE: AGE receptor
rRNA: ribosomal ribonucleic acid
SRM: selective-reaction monitoring
