# Supplementary figures and images for: Heat-treated high-fat diet modifies gut microbiota and metabolic markers in apoe−/− mice
Source: Nutr Metab (Lond). 2016 Mar 12;13:22. doi: 10.1186/s12986-016-0083-0 (PMC4788960; doi:10.1186/s12986-016-0083-0)

**High-fat diet**

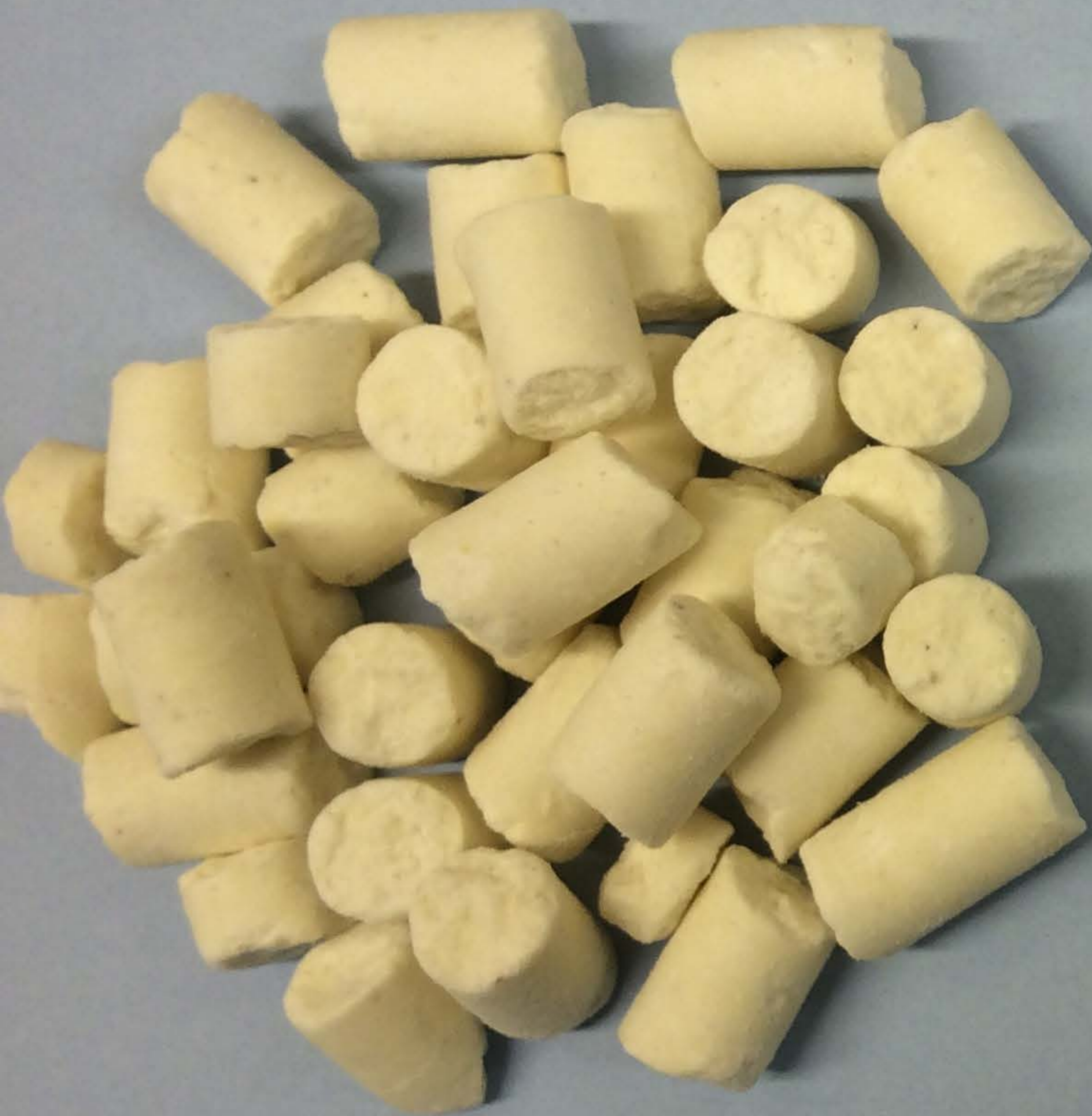

**Heat-treated high-fat diet**

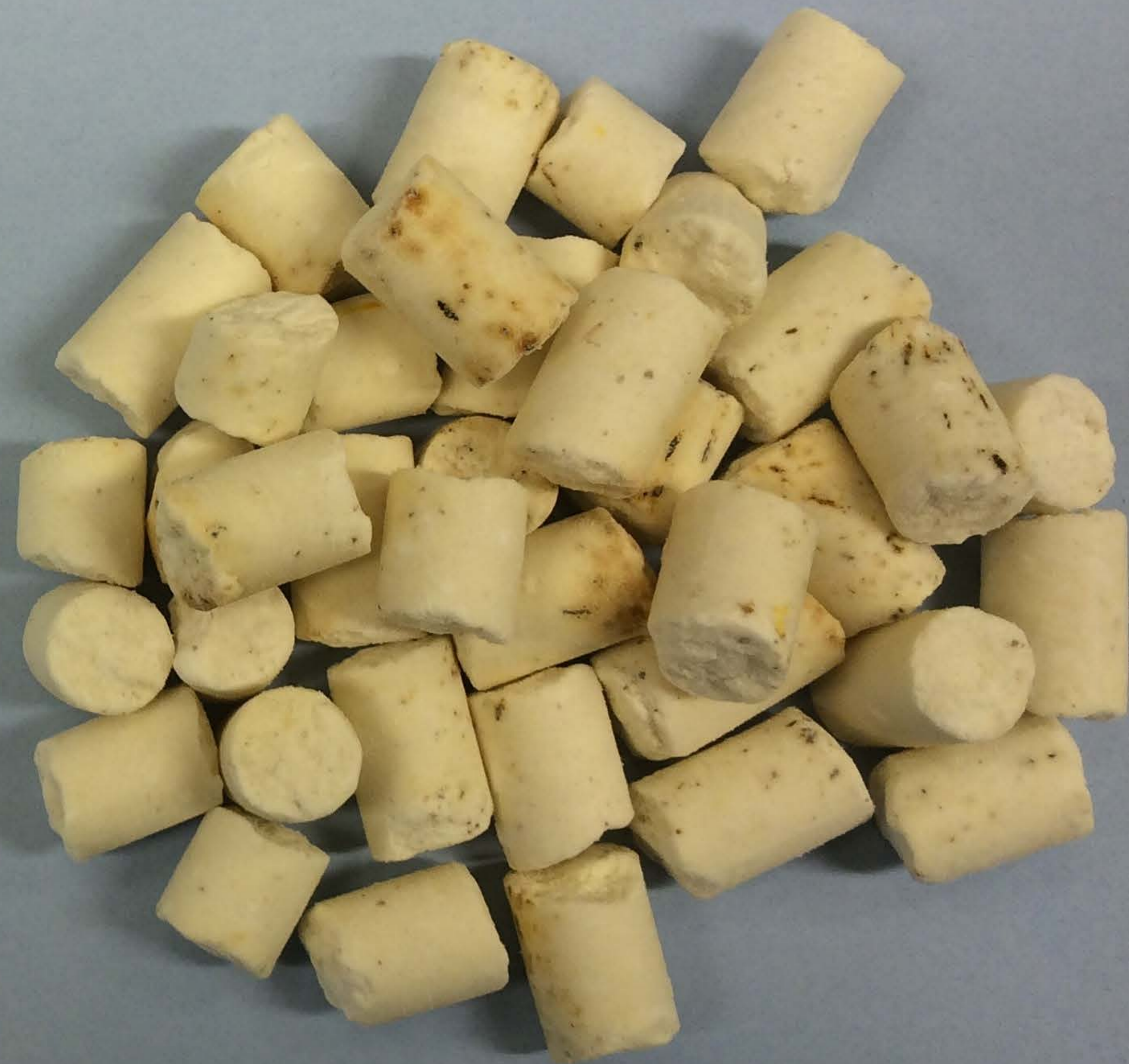

Supplement: Additional file 1: Figure S1. — Unheated (left) and heat-treated (200 °C for 10 min, right) high-fat diets. (PDF 241 kb) [file 12986_2016_83_MOESM1_ESM.pdf]
