# Supplementary material for: Heat-treated high-fat diet modifies gut microbiota and metabolic markers in apoe−/− mice
Source: Nutr Metab (Lond). 2016 Mar 12;13:22. doi: 10.1186/s12986-016-0083-0 (PMC4788960; doi:10.1186/s12986-016-0083-0)
Supplement: Additional file 2: Figure S2. — Cladogram plot showing bacterial taxa with LDA scores higher than two using strict version of LEfSe. Different colors represent the most abundant taxa in different groups of mice (yellow indicating LF, red indicating HF and blue indicating HT). Circles represent phylogenetic levels from phylum to genus. The sizes of the circle are proportional to the taxon’s abundance. (PDF 436 kb) [file 12986_2016_83_MOESM2_ESM.pdf]

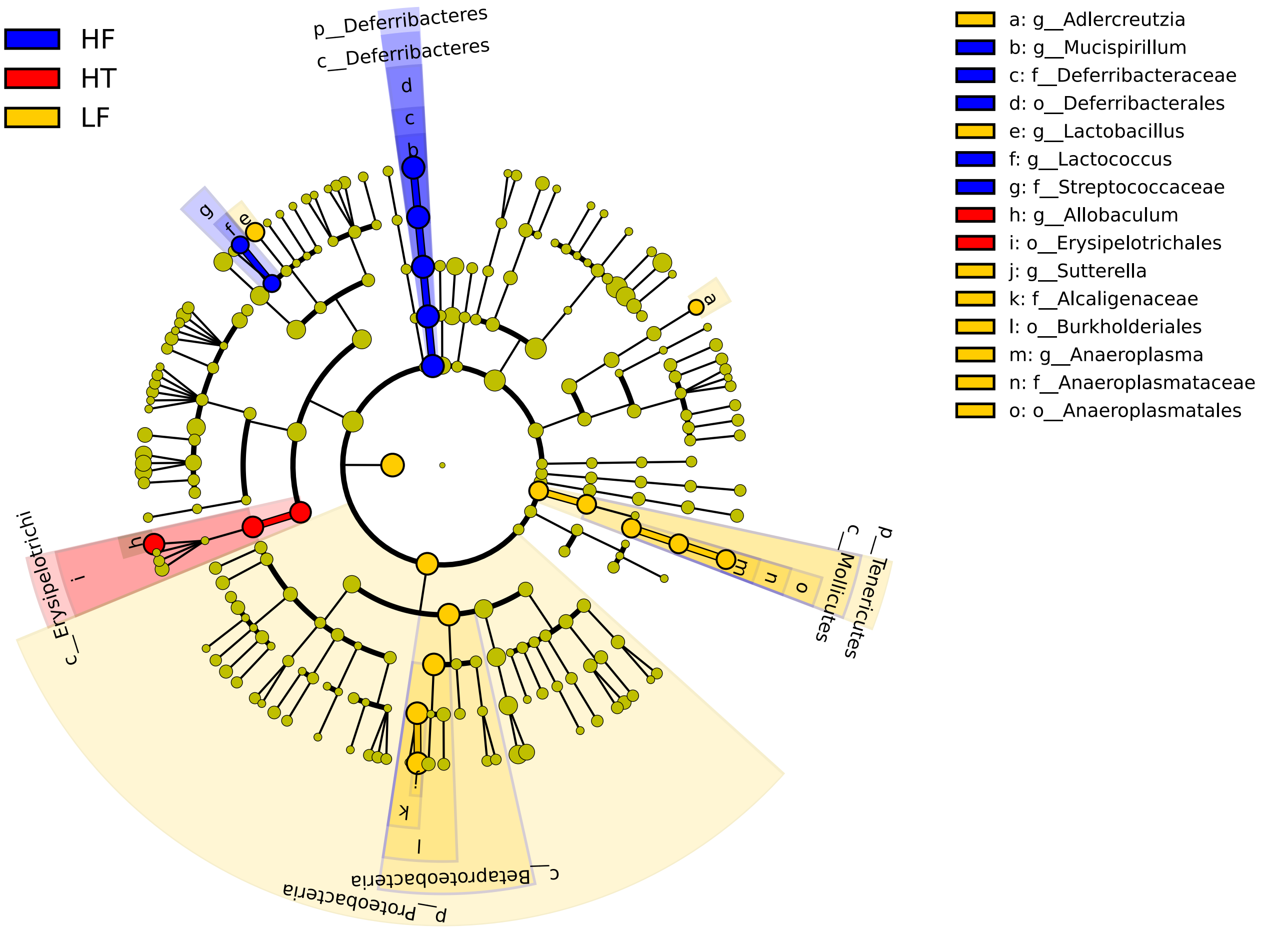
HF
HT
LF
p__Deferribacteres
c__Deferribacteres
c__Erysipelotrichi
p__Tenericutes
c__Mollicutes
c__Betaproteobacteria
p__Proteobacteria
a: g__Adlercreutzia
b: g__Mucispirillum
c: f__Deferribacteraceae
d: o__Deferribacterales
e: g__Lactobacillus
f: g__Lactococcus
g: f__Streptococcaceae
h: g__Allobaculum
i: o__Erysipelotrichales
j: g__Sutterella
k: f__Alcaligenaceae
l: o__Burkholderiales
m: g__Anaeroplasma
n: f__Anaeroplasmataceae
o: o__Anaeroplasmatales
